# A Cross-National Analysis of the Effects by Bullying and School Exclusion on Subjective Happiness in 10-Year-Old Children

**DOI:** 10.3390/children9020287

**Published:** 2022-02-18

**Authors:** Diego Gomez-Baya, Francisco Jose Garcia-Moro, Javier Augusto Nicoletti, Rocio Lago-Urbano

**Affiliations:** 1 Department of Social, Developmental and Educational Psychology, Universidad de Huelva, 21007 Huelva, Spain; diego.gomez@dpee.uhu.es (D.G.-B.); fjose.garcia@dpsi.uhu.es (F.J.G.-M.); 2 Department of Humanities and Social Sciences, Universidad Nacional de La Matanza, San Justo, Buenos Aires B1754, Argentina; javiernicoletti@gmail.com

**Keywords:** bullying, exclusion, happiness, well-being, children, cross-national, cross-sectional

## Abstract

Literature to date has well supported the detrimental consequences of bullying and school exclusion in different countries, with negative outcomes in school adjustment or child psychological adjustment, among others. However, more research is needed to understand the effects on positive indicators of psychological well-being in children as subjective happiness. Cross-national studies are also recommended to examine the differential effects by country. Thus, the aim of this study was to examine bullying and school exclusion, and their effects on child subjective happiness, from a cross-national perspective. Data from the Second Wave of Children’s Worlds: International Survey of Children’s Well-being (ISCWeB) was used, from a sample of 12,623 children aged 10 years old from 15 countries. Participants completed self-report measures of bullying, school exclusion and subjective happiness. Results showed that 20.8% of children suffered harassment and 17.6% felt excluded, twice or more times, at school. Negative effects of bullying and exclusion on subjective happiness were observed in all the sample. Furthermore, differences by country were found in the frequency of bullying and exclusion, as well as in the size of their effects on happiness. These results underline the need to protect child psychological well-being by preventing bullying and school exclusion.

## 1. Introduction

The universal education of children is a right and a duty accepted and carried out, in practice, by the majority of countries. Efforts being made to ensure that more and more children receive a quality education that will allow them to develop adequately and provide constructive answers to the problems that arise in our globalized civilization must be accompanied by actions that guarantee a positive climate, allowing their healthy development in educational institutions; in this sense, although it is true that the school climate in our educational centers favors a healthy development of students [1,2], it is also true that one of the most important threats that upsets the welfare of our students is the phenomenon of bullying that they may suffer throughout their schooling. This reality requires cross-national research to shed light on this situation, as well as to analyze progress towards the goal of providing a safe, stable learning environment that promotes effective learning [3]. In this sense, the concept of subjective well-being is central for our research, which refers to a person’s subjective evaluation of the quality of his or her life [4]. Its study comprises the scientific analysis of how people evaluate their lives in a specific or general manner [5].

Bullying, as established by Olweus [6], refers to a particularly serious form of aggression in which one or more aggressor students exercise some type of violence, continued over time, towards another student who is at a disadvantage or an inferior position with respect to the aggressor, due to lack of social support from peers, personality characteristics, etc. [7] Bullying includes physical, psychological and sexual aggression and occurs mostly between peers, but, in some cases, from teachers and other school staff [3]. 

The reality of bullying is very problematic for child development, and the literature to date has documented its alarming prevalence; various studies place at between 10% and 33% the percentage of pupils who are victims of bullying [8]. According to the UNESCO report [3], approximately 32% of students have been bullied by their peers at school on one or more days in the month before the research. There are significant differences between regions. The proportion of students reporting that they have been bullied is highest in the Middle East, North Africa, and Sub-Saharan Africa and lowest in Central America, the Caribbean, and Europe. On their part, meta-analyses of 80 studies analyzing rates of participation in bullying (both bullying others and being bullied) for students aged 12–18 years reported a mean prevalence rate of 35% for participation in traditional bullying and 15% for participation in cyberbullying [9]. Patchin & Hinduja [10] indicate that 49.8% of preadolescent (9 to 12 years old) said they had experienced bullying at school and 14.5% of preadolescent stated that they had experienced cyberbullying.

With regard to the consequences of being a victim of bullying, research yields very worrying data. In this regard, it can lead to depressive and anxiety symptoms [11,12,13], and the bullied are twice as likely as their non-bullied peers to experience negative health effects, such as headaches and stomachaches [14], exclusionary situations [15,16,17,18,19], difficulties in academic performance [20], sleeping difficulties, lower academic performance and dropping out of school [21], are more prone to depression, prolonged victimization and maladjustment [22], and can even lead to suicidal ideation, suicide attempts or even completed suicides [23]. Negative effects on subjective well-being are also demonstrated in different studies [24,25].

Research also yields important data regarding protective factors within the school context. Thus, McCallion and Feder [26] indicate that bullying prevention programs decrease such behavior by up to 25%. In turn, ref. [27] report the importance of peer support to overcome situations as self-reported by bullied students; along these lines, ref. [28] also point out that peer support programs, such as peer mediation in elementary schools, which consists of training students as mediators in other children’s conflicts by encouraging them to talk about their feelings and reach solutions to address bullying or other problems, could be used to help prevent a child from being excluded. On their part, [27] refer to the importance given by the student victim of bullying to the figure of the teacher, and to his or her way of proceeding, as in getting positively involved in solving the problem by listening and checking for problem behaviors.

Despite that the literature to date has clearly established the detrimental consequences of bullying and school exclusion on child adjustment, underlining many negative outcomes as consequences, more evidence is needed concerning the negative effects on positive indicators of psychological well-being as subjective happiness. Because mental health entails both the absence of problems and the presence of psychological well-being, more research is recommended to examine the consequences on this positive side of mental health in childhood. It would be interesting to deepen research on bullying and exclusion from the perspective of subjective happiness, in order to complement the evidence with regards to other important results, such as the consequences it produces, e.g., stress, depression or anxiety. Different research projects in the field of subjective well-being have emphasized the importance of arriving at cognitive judgments about the degree of satisfaction with life, but with a more global dimension; that is, the adjustment of the person within the social and environmental context, the school being one of the most significant examples [29,30,31]. Moreover, because most studies to date have addressed samples from different countries, more research is recommended to integrate data from a cross-national perspective. Thus, the aims of the present work were: (a) to explore the frequency of bullying and exclusion in 10-year-old children, and (b) to examine the effects of bullying and exclusion experiences on subjective happiness from an international perspective. 

## 2. Materials and Methods

### 2.1. Data Collection Procedure

The present work used secondary data from the Second Wave of Children’s Worlds: International Survey of Children’s Well-being (ISCWeB), collected in 2013–2014 and published in 2016. This survey collected data to examine self-perceptions of children about their psychological well-being and their assessment of their lives in different developmental contexts (such as peers, family and school), from a cross-national perspective. The data are available by request for academic purposes at www.isciweb.org (accessed on 8 January 2022). The authors of the present article completed the appropriate formal request and were adequately authorized. 

This study followed a cross-sectional and descriptive design and data were collected from 15 countries (Algeria, Nepal, Estonia, Spain, Colombia, Turkey, Ethiopia, South Korea, Germany, Israel, Romania, Norway, Poland, South Africa, and Malta), during the winter of 2013 and the spring of 2014. For the purpose of the present work, only data from participants aged 10 years old were examined. Ethical permission from the appropriate ethics boards in each country was approved, and parental informed consents were collected. This procedure respected the privacy, anonymity and confidentiality of the participants. For further information concerning sampling procedure in each country, the national reports were published on the website (https://isciweb.org/the-data/publications/country-reports/country-reports-of-thesecond-wave-2013-2014/). 

### 2.2. Participants

For the purpose of this study, we examined data from a total of 12,623 children aged 10 years old (50.1% girls) from 15 countries (Algeria: 6.2%, Nepal: 7.8%, Estonia: 6.8%, Spain: 7%, Colombia: 7.3%, Turkey: 7.1%, Ethiopia: 4.4%, South Korea: 19.3%, Germany: 3.6%, Israel: 3.2%, Romania: 8.2%, Norway: 5%, Poland: 4.6%, South Africa: 8.4%, and Malta: 1%). Sample composition by gender and country is presented in Table 1. Up to 97.5% of the participants were born in the same country in which they lived. In each country, a representative sampling procedure was followed. Moreover, 98.4% of the sample lived with their families, and most of the sample always (64.1%) or usually (31.2%) slept in the same home. All these children were enrolled in a school selected for the study, provided the respective informed consent, and attended class on the fieldwork day.

### 2.3. Instrument

The sample of participants filled in self-report measures of well-being and of different characteristics of their lives and their developmental contexts. In each country, the scales were back translated from English to each native language. For the purposes of this study, only measures for demographics (gender and nationality), bullying and school exclusion, and subjective happiness were described. In order to assess school bullying and exclusion two questions were used: “How often, if at all, in the last month have you been: Hit by other children in your school?/Left out by other children in your class?”, with 4 response options, i.e., never (0), once (1), 2–3 times (2), and more than 3 times (3). The question “Overall, how happy have you been feeling during the last two weeks?” was used to assess subjective happiness, with a scale from 0 (not at all happy) to 10 (totally happy).

### 2.4. Data Analysis Design

First, the descriptive statistics (i.e., mean and standard deviation) of subjective happiness and the frequency distribution of the separate items of school bullying and exclusion were examined in the total sample and by country. Differences by gender and country were calculated in subjective happiness by conducting Student *t*-test and variance analysis. Differences by gender and country in school bullying and exclusion were examined with χ^2^ tests. Second, variance analyses were performed to examine the relationships between school bullying and exclusion and the scores for subjective happiness in the total sample and by country. Third, stepwise regression analyses were conducted to examine subjective happiness based on the demographics in step 1 (i.e., gender and nationality) and separate indicators of school bullying and school exclusion in step 2. Then, this analysis was conducted by country. F, R^2^, and standardized coefficients (β) were reported. Multicollinearity and self-correlation (Durbin–Watson test) were calculated. All these analyses were performed with SPSS 21.0 program (IBM Corp., Armonk, NY, USA).

## 3. Results

### 3.1. Descriptive Statistics of Study Variables

Table 2 shows the mean and standard deviation of subjective happiness and school bullying and exclusion, in the total sample and by gender. Concerning subjective happiness, a high mean score was observed in the overall sample, indicating that 10-year-old children in the study were on average very happy. Significant differences in subjective happiness were observed by country, F(14, 11,242) = 3.81, *p* < 0.001, partial eta squared = 0.040. The highest mean scores were observed in Romania and Turkey, while the lowest were detected in Estonia and South Korea. No differences were observed by gender, t(12,442) = −0.01, *p* = 0.995. With regards to school bullying and exclusion, the overall mean scores were apparently low, indicating a score below 1, which represents less than one experience of bullying or exclusion. No differences by nationality were observed in happiness, F(1, 12,432) = 0.22, *p* = 0.637, but children from another nationality than that of the country surveyed reported more bullying, F(1, 12,144) = 12.35, *p* < 0.001, partial eta squared = 0.001, and exclusion, F(1, 12,005) = 21.94, *p* < 0.001, partial eta squared = 0.002. Moreover, children who always sleep in the same home were happier, F(2, 12,240) = 12.32, *p* < 0.001, partial eta squared = 0.002, and reported less bullying, F(2, 11,962) = 29.16, *p* < 0.001, partial eta squared = 0.005, and exclusion, F(2, 11,824) = 35.09, *p* < 0.001, partial eta squared = 0.006. 

**Figure 1:**
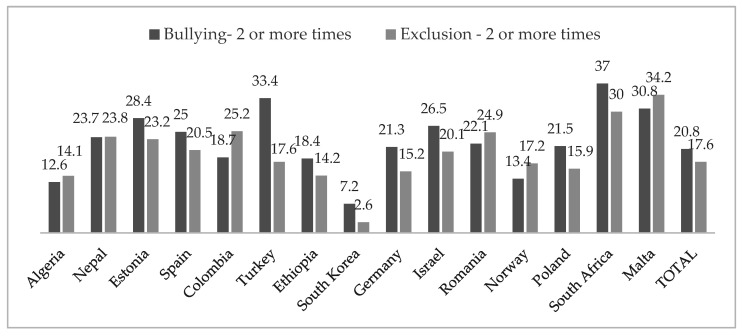
Percentage of participants who experienced, twice or more times, bullying and exclusion in the total sample and by country.

When examining the percentage of children who experienced bullying or exclusion twice or more, the results were more problematic (see Figure 1). Results pointed out that 20.8% of the total sample suffered bullying, and 17.6% of the sample suffered exclusion at school. Furthermore, 62.8% of the sample indicated no bullying, while 16.4% indicated one experience of harassment. Regarding school exclusion, 67.9% reported no exclusion and 14.5% indicated that they felt excluded once. Differences by country were observed in bullying, χ^2^ (28, *N* = 12,155) = 970.47, *p* < 0.001, φ = 0.28, and in exclusion, χ^2^ (28, *N* = 12,016) = 1272.60, *p* < 0.001, φ = 0.33. The countries with the greatest percentages for bullying were South Africa, Turkey and Malta, while the countries with the lowest percentages were South Korea, Algeria and Norway. Furthermore, the countries with more school exclusion were Malta, South Africa and Colombia, while the countries with less exclusion were South Korea, Algeria and Ethiopia. Finally, concerning gender differences in the overall sample in bullying and exclusion, results detected that 25.6% of the boys’ sample suffered bullying, compared to 16% of the girls’ sample, χ^2^ (2, *N* = 12,155) = 213.60, *p* < 0.001, φ = 0.13. No differences by gender were found in exclusion, χ^2^ (2, *N* = 12,016) = 4.16, *p* = 0.125, φ = 0.02. 

### 3.2. Associations between Bullying, School Exclusion and Subjective Happiness

Table 3 and Table 4 describe the differences in the level of subjective happiness for bullying and school exclusion, respectively, in both the total sample and each country. First, results showed that children who suffered bullying (two or more times) reported less subjective happiness than those who did not suffer any bullying, across the total sample. The strongest effects for bullying on child happiness were observed in Germany, Malta and Norway, while the smallest effects were detected in Nepal, Colombia and South Africa. Second, results also indicated that children who suffered exclusion at school (two or more times) reported less happiness than those who did not. When comparing the results by country, some differences were discovered. The biggest effects were found in Poland, Norway and Germany, while the smallest ones were observed in Romania, Colombia and Nepal. No gender moderation was observed either in bullying, β = −0.003, *p* = 0.777, or in exclusion, β = −0.013, *p* = 0.180.

### 3.3. Regression Analyses to Examine the Combined Effects of School Bullying and Exclusion on Child Happiness

**Figure 2:**
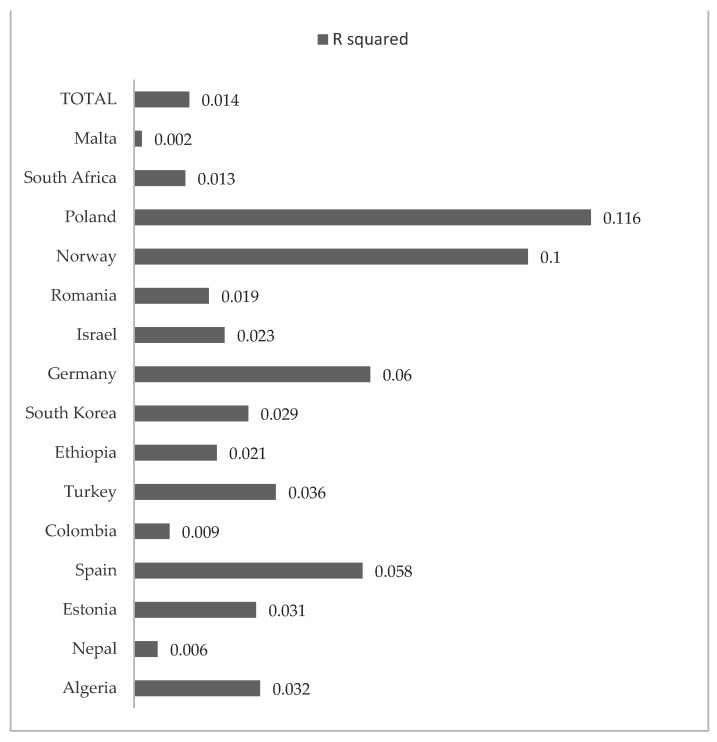
R^2^ values in the regression analyses to explain subjective happiness on the basis of bullying and exclusion, in the total sample and by country.

Table 5 describes the results of the regression analyses in the total sample and by country to examine the effects of bullying and exclusion on child happiness. In the total sample, both bullying and exclusion had negative effects on child happiness. These effects were significant, although small in size. The explained variance of child happiness in the total sample was small (1.4%). Significant small negative effects of bullying were detected in Estonia, Spain, Ethiopia, South Korea, Germany, Romania, Norway and Poland. Significant negative effects of exclusion were found in all countries, except in Romania and Malta. Specifically, moderate effects were found in Poland and Norway. Figure 2 represents the R^2^ values of the regression analyses, also performed by country. More explained variance of child happiness based on bullying and exclusion was found in Poland, Norway and Germany, and lower scores were observed in Malta, Nepal and Colombia. 

## 4. Discussion

Bullying and exclusion from school is a reality in the lives of many children, and given its importance, numerous experts have examined this behavior more closely to determine the cause of bullying, how it can be modified, what are its consequences [32] or what role families play [33,34], among other aspects. In this sense, the aim of the present study was to analyze the frequency of exclusion and bullying in 10-year-old children. More specifically, we aimed to explore the effects of the experiences of exclusion and bullying on subjective happiness with a cross-national approach, taking into account that it would be interesting to deepen research on bullying from the perspective of subjective happiness, in addition to other important factors such as the consequences it produces, such as stress, depression or anxiety.

First, the data on the 10-year-old children participating in the study reflect a high average score for subjective happiness. That is, they are very happy children. As indicated in the results section, there are significant differences by country that could be explained by idiosyncratic variables, with impact on the quality of other developmental contexts, such as the family. 

The data from this study revealed that only 62.8% of 10-year-olds in the participating countries had never been hit by other children at school, whereas 20.8% had had this experience on two or more occasions in the last month. This number is similar to that provided in the investigations collected in the meta-analysis carried out by Modecki [9]. In relation to school exclusion, only 67.9% of the children had never been left out by others, while 17.6% suffered it on two or more occasions in the last month. According to the scientific literature [15,16,17,18,19], school exclusion is increasingly recognized as relevant to children’s health, existing risk factors including children who are looked after, children with special educational needs, those living in poverty, or from some ethnic minorities. From a cross-national perspective, our results also revealed high that frequencies in school exclusion and bullying were observed in South Africa, Turkey and Malta, and lower frequencies in South Korea, Algeria and Norway.

In addition, in the general sample, both exclusion and bullying experiences presented a negative impact on children’s happiness. These results are in line with those obtained previously which state that minors who feel unsafe in their school are victims of bullying and tend to report lower levels of subjective well-being [35,36]. Furthermore, this is associated with greater personal insecurity, anxiety, depression, loneliness and unhappiness [37]. Specifically in the research we present, higher effects of school exclusion and bullying on happiness were found in Poland, Norway and Germany, while smaller effects were observed in Malta, Nepal and Colombia. These differences in size effects may be due to possible differences in violence exposure in these countries, what could make varied the concrete influence of these negative school experiences on child happiness. The differential effects of bullying and exclusion on happiness may be also explained by the different determinants of peer violence in each country and the joint effect with other remarkable factors, such as the quality of overall living conditions, the quality of school context or the existence of specific educational policies to protect child development. 

Likewise, the results of this work showed that a high percentage of children under 10 years of age in 15 countries from different continents acknowledged the experience of school exclusion and bullying during the last month, and the results underlined that these experiences had a negative impact on child happiness. Previous studies have found that having been a victim is not only highly correlated with having higher levels of depression and interpersonal anxiety, but also with having low self-esteem and feeling more insecure about support received from their social environment [38]. On their part, other studies report that victims of school violence have difficulties relating to their peers [39], difficulties in academic performance [20], difficulties falling asleep, lower academic performance and school dropout [21], all aspects that have an effect on people’s subjective happiness. 

Children who experience both bullying and school exclusion are less happy. In general, negative effects of exclusion and bullying on subjective happiness were observed in the entire sample. Taking into account the differences found between countries in both bullying and exclusion, and their impact on children’s happiness, the design of prevention programs must take into account the different risk and protection factors for each country, and direct attention to protecting the child mental health taking into account the particular characteristics of each school context and academic system. Previous works regarding the application of intervention programs based on bullying prevention provide key ideas for creating programs of coexistence within schools; an example of this is the “Buen Trato” [Well treated] program [40] that has been developed in ANAR-Peru since 2007 and has been in operation for 6 years in Spain. UNICEF—United Nations recognized this program in 2010 as being an example of “Good Practices for Child Participation in the defense of children’s rights”. This program focuses on providing training in values and basic skills to combat violence to volunteer students so that they in turn become “trainers” of their younger classmates, becoming role models for them, and fostering good treatment that facilitates coexistence between equals. Another example focusing on the prevention of bullying is the KIVA method, where all students assume the responsibility not to encourage harassment and to support victims. This method has been shown to be effective in preventing bullying, cyberbullying and other types of school violence in children 6 to 12 years old [41]. Moreover, a positive education paradigm could be recommendable to guide the design of intervention programs in order to promote psychological well-being in the school context [42]. For this purpose, it is essential to rethink the concept of education, teaching methods and school organization itself, as well as highlighting subjective happiness as one of the key objectives within study plans [43,44]. A good example of this line of intervention is the whole-school experience in Geelong Grammar school in Australia, integrating the promotion of well-being and character strengths in the teaching procedures for academic development [45]. Another example of good practice focused on positive youth development is the Lights4Violence international educational intervention project [46] that promotes work in positive relationships between adolescents, based on the strengths of adolescents themselves. This project tries to promote protective factors to prevent gender violence, focusing on assets such as empathy, communication skills, prosocial skills or non-violent conflict resolution, among others.

This study has strengths and limitations. Regarding strengths, this research comprised data from a sizeable sample from 15 countries (Algeria, Nepal, Estonia, Spain, Colombia, Turkey, Ethiopia, South Korea, Germany, Israel, Romania, Norway, Poland, South Africa and Malta). Anther strength is the separate and joint analysis of bullying and school exclusion and their respective effects on child happiness. Regarding the limitations, it is important to take into account that it is a cross-sectional study, and the conclusions can only be based on bidirectional associations between the variables. A longitudinal study would be recommended to analyze prospective effects of exclusion and bullying on child happiness. Moreover, since self-report measures were used, results may be biased by social desirability. More objective indicators and multiple informants would be necessary to reach a more valid assessment. Another limitation in this study was the consideration of a specific age in the sample, in our case children 10 years old, so in future studies data for different ages throughout childhood and adolescence could be examined. In any case, the examination of children’s well-being and its determinants around the world is an important contribution to the literature from the international project Children’s Worlds, which may complement the data on subjective happiness collected from more general samples, such as the project behind the World Happiness Report [47].

Finally, these results indicate the need to consider lines of research focused on knowing the influence that bullying and school exclusion have on the subjective happiness of minors and the need to design and develop preventive programs from a global and ecological perspective that include minors, the family and the educational context.

## 5. Conclusions

The present research showed that, in the total sample from 15 countries, around one fifth of the children suffered harassment and around one sixth had felt excluded, twice or more times, at school. Negative effects of bullying and exclusion on subjective happiness were observed in all the sample. Furthermore, differences by country were found in the frequency of bullying and exclusion, as well as in the size of their effects on happiness. These results underline the need to protect child psychological well-being by preventing bullying and school exclusion.

## Figures and Tables

**Table 1:** Sample composition by gender and country.

	Boys %(*n*)	Girls %(*n*)	Total *n*
Algeria	49.4 (385)	50.6 (395)	780
Nepal	49.7 (489)	50.3 (494)	983
Estonia	51.6 (441)	48.4 (413)	854
Spain	50.2 (445)	49.8 (441)	886
Colombia	47.9 (444)	52.1 (483)	927
Turkey	50.4 (450)	49.6 (443)	893
Ethiopia	49 (274)	51 (285)	559
South Korea	48.9 (1192)	51.1 (1246)	2438
Germany	48 (220)	52 (238)	458
Israel	49.9 (204)	50.1 (205)	409
Romania	52.8 (546)	47.2 (489)	1035
Norway	51.3 (326)	48.7 (309)	635
Poland	51.4 (300)	48.6 (284)	584
South Africa	48.4 (513)	51.6 (548)	1061
Malta	62 (75)	38 (46)	121
Total	49.9 (6304)	50.1 (6319)	12,623

**Table 2:** Mean and standard deviation of study variables in the total sample and by country.

	Subjective Happiness 0–10	School Bullying 0–3	School Exclusion 0–3
Algeria	8.87 (2.03)	0.48 (0.85)	0.57 (0.90)
Nepal	8.64 (2.07)	0.82 (1.02)	0.86 (1.01)
Estonia	8.16 (2.09)	0.91 (1.13)	0.76 (1.02)
Spain	9.06 (1.57)	0.79 (1.08)	0.67 (0.97)
Colombia	9.20 (1.71)	0.62 (1.00)	0.83 (1.11)
Turkey	9.33 (1.77)	1.09 (1.14)	0.55 (0.99)
Ethiopia	8.61 (2.08)	0.62 (0.96)	0.47 (0.88)
South Korea	8.24 (2.05)	0.27 (0.69)	0.10 (0.44)
Germany	8.32 (1.97)	0.67 (1.00)	0.53 (0.93)
Israel	8.57 (2.39)	0.84 (1.07)	0.70 (1.08)
Romania	9.35 (1.46)	0.69 (1.00)	0.78 (1.13)
Norway	8.86 (1.91)	0.48 (0.84)	0.58 (0.93)
Poland	8.86 (1.65)	0.69 (1.04)	0.53 (0.93)
South Africa	8.67 (2.35)	1.17 (1.25)	0.96 (1.18)
Malta	8.50 (2.12)	1.03 (1.14)	1.08 (1.18)
Total	8.73 (1.99)	0.69 (1.03)	0.59 (0.98)

**Table 3:** Relationship between bullying and subjective happiness in the total sample and by country.

	F	Partial eta Squared	Never M(SD)	Once M(SD)	2 or More Times M(SD)
Algeria	2.76	0.007	8.96 (1.94)	8.90 (1.54)	8.43 (2.78)
Nepal	0.49	0.001	8.62 (2.05)	8.78 (1.94)	8.61 (2.17)
Estonia	12.47 ***	0.030	8.45 (1.81)	8.30 (1.97)	7.62 (2.50)
Spain	11.67 ***	0.027	9.26 (1.31)	8.99 (1.72)	8.67 (1.82)
Colombia	1.45	0.003	9.24 (1.74)	9.22 (1.46)	8.99 (1.82)
Turkey	8.52 ***	0.019	9.55 (1.58)	9.42 (1.53)	8.99 (2.08)
Ethiopia	5.78 **	0.021	8.83 (1.88)	8.19 (2.36)	8.24 (2.35)
South Korea	29.63 ***	0.025	8.40 (1.94)	7.87 (2.18)	7.25 (2.60)
Germany	14.91 ***	0.067	8.73 (1.67)	7.60 (2.19)	7.73 (2.33)
Israel	1.49	0.008	8.75 (2.21)	8.51 (2.45)	8.25 (2.64)
Romania	9.68 ***	0.019	9.50 (1.31)	9.30 (1.32)	9.00 (1.89)
Norway	17.83 ***	0.057	9.18 (1.62)	8.24 (2.29)	8.15 (2.31)
Poland	14.02 ***	0.051	9.12 (1.32)	8.82 (1.60)	8.20 (2.21)
South Africa	3.17 *	0.006	8.87 (2.14)	8.50 (2.35)	8.50 (2.58)
Malta	3.59 *	0.059	8.94 (1.45)	7.64 (2.25)	8.47 (2.66)
Total	52.16 ***	0.009	8.87 (1.84)	8.66 (1.99)	8.42 (2.35)

Note. *** *p* < 0.001; ** *p* < 0.01; * *p* < 0.05.

**Table 4:** Relationship between school exclusion and subjective happiness in the total sample and by country.

	F	Partial eta Squared	Never M(SD)	Once M(SD)	2 or More Times M(SD)
Algeria	16.31 ***	0.042	9.09 (1.72)	8.95 (1.76)	7.89 (3.05)
Nepal	4.70 **	0.012	8.81 (1.82)	8.68 (2.12)	8.25 (2.45)
Estonia	9.91 ***	0.024	8.47 (1.85)	7.82 (2.17)	7.81 (2.37)
Spain	21.36 ***	0.050	9.32 (1.30)	8.95 (1.28)	8.44 (2.18)
Colombia	4.62 *	0.011	9.31 (1.65)	9.30 (1.53)	8.90 (1.89)
Turkey	17.38 ***	0.039	9.56 (1.40)	8.94 (2.26)	8.75 (2.25)
Ethiopia	5.07 **	0.018	8.78 (1.92)	8.13 (2.45)	8.16 (2.37)
South Korea	22.58 ***	0.019	8.36 (1.97)	7.33 (2.21)	7.08 (2.84)
Germany	11.60 ***	0.053	8.63 (1.66)	8.10 (1.84)	7.42 (2.69)
Israel	5.24 **	0.027	8.85 (1.88)	8.02 (2.96)	8.04 (3.21)
Romania	0.42	0.001	9.38 (1.51)	9.25 (1.51)	9.34 (1.31)
Norway	27.86 ***	0.087	9.25 (1.49)	8.69 (1.89)	7.78 (2.59)
Poland	31.16 ***	0.111	9.23 (1.13)	8.57 (1.66)	7.81 (2.50)
South Africa	7.74 ***	0.014	8.93 (2.04)	8.44 (2.60)	8.33 (2.66)
Malta	1.14	0.020	8.79 (1.78)	8.09 (2.25)	8.26 (2.51)
Total	70.72 ***	0.012	8.89 (1.80)	8.59 (2.09)	8.34 (2.43)

Note. *** *p* < 0.001; ** *p* < 0.01; * *p* < 0.05.

**Table 5:** Regression analysis to explain subjective happiness based on bullying and exclusion, in the total sample and by country.

		Bullying		Exclusion	
	F	β	*t*	β	*t*
Algeria	13.40 ***	−0.02	−0.50	−0.18 ***	−4.75
Nepal	3.27 *	0.05	1.16	−0.10 *	−2.55
Estonia	13.19 ***	−0.13 **	−3.33	−0.08 *	−2.06
Spain	25.41 ***	−0.12 **	−3.40	−0.18 ***	−5.09
Colombia	4.70 **	−0.02	−0.60	−0.10 **	−2.60
Turkey	17.09 ***	−0.07	−1.89	−0.16 ***	−4.37
Ethiopia	6.81 **	−0.10 *	−2.19	−0.09 *	−2.03
South Korea	35.61 ***	−0.13 ***	−5.88	−0.08 ***	−3.61
Germany	13.95 ***	−0.15 **	−2.66	−0.15 **	−2.77
Israel	5.30 **	−0.07	−1.25	−0.14 **	−2.70
Romania	10.45 ***	−0.15 ***	−4.52	0.02	0.52
Norway	32.87 ***	−0.13 **	−3.14	−0.25 ***	−6.06
Poland	33.00 ***	−0.10 *	−2.14	−0.29 ***	−6.12
South Africa	8.07 ***	−0.04	−1.27	−0.11 **	−3.30
Malta	1.13	−0.08	−0.84	−0.09	−0.93
Total	85.79 ***	−0.06 ***	−6.20	−0.08 ***	−8.41

Note. *** *p* < 0.001; ** *p* < 0.01; * *p* < 0.05.

## Data Availability

Data are accessible on the website www.isciweb.org (accessed on 8 January 2022).

## References

[1] Alfaro J., Guzmán J., Reyes F., García C., Varela J., Sirlopú D. (2016). Satisfacción global con la vida y satisfacción escolar en estudiantes Chilenos [Overall life satisfaction and school satisfaction in Chilean students]. Psykhe Rev. De La Esc. De Psicol..

[2] García Bacete F.-J., Sureda García I., Monjas Casares M.I. (2010). El rechazo entre iguales en la educación primaria: Una panorámica general. An. Psicol./Ann. Psychol..

[3] UNESCO (2018). School Violence and Bullying: Global Status and Trends, Drivers and Consequences. http://www.infocoponline.es/pdf/BULLYING.pdf.

[4] Bender T.A. (1996). Assessment of Subjective Well-Being during Childhood and Adolescence. Handbook of Classroom Assessment.

[5] Diener E., Oishi S., Lucas R.E. (2003). Personality, culture, and subjective well being: Emotional and cognitive evaluations of life. Annu. Rev. Psychol..

[6] Olweus D. (1993). Bullying at School: What We Know and What We Can Do.

[7] Suárez-García Z., Álvarez-García D., y Rodríguez C. (2020). Predictores de ser víctima de acoso escolar en Educación Primaria: Una revisión sistemática. Rev. De Psicol. Y Educ..

[8] Hymel S., Swearer S.M. (2015). Four Decades of Research on School Bullying. Am. Psychol..

[9] Modecki K.L., Minchin J., Harbaugh A.G., Guerra N.G., Runions K.C. (2014). Bullying prevalence across contexts: A meta-analysis measuring cyber and traditional bullying. J. Adolesc. Health.

[10] Patchin J.W., Hinduja S. (2020). Tween Cyberbullying in 2020. Cyberbullying Research Center and Cartoon Network. https://i.cartoonnetwork.com/stop-bullying/pdfs/CN_Stop_Bullying_Cyber_Bullying_Report_9.30.20.pdf.

[11] Graham S., Bellmore A.D., Mize J. (2006). Peer Victimization, Aggression, and Their Co-Occurrence in Middle School: Pathways to Adjustment Problems. J. Abnorm. Child Psychol..

[12] Kerr J.C., Valois R.F., Huebner E.S., Drane J.W. (2011). Life Satisfaction and Peer Victimization among USA Public High School Adolescents. Child Ind. Res..

[13] Estévez E., Murgui S., Musitu G. (2009). Psychological adjustment in bullies and victims of school violence. Eur. J. Psychol. Educ..

[14] Gini G., Pozzoli T. (2013). Bullied children and psychosomatic problems: A meta-analysis. Pediatrics.

[15] Paget A., Parker C., Heron J., Logan S., Henley W., Emond A., Ford T. (2018). Which children and young people are excluded from school? Findings from a large British birth cohort study, the Avon Longitudinal Study of Parents and Children (ALSPAC). Child Care Health Dev..

[16] Sproston K., Sedgewick F., Crane L. (2017). Autistic girls and school exclusion: Perspectives of students and their parents. Autism Dev. Lang. Impair..

[17] Hodge N., Wolstenholme C. (2016). ‘I didn’t stand a chance’: How parents experience the exclusions appeal tribunal. Int. J. Incl. Educ..

[18] Levinson M. (2016). ‘I don’t need pink hair here’: Should we be seeking to ‘reintegrate’ youngsters without challenging mainstream school cultures?. Int. J. Sch. Disaffection.

[19] Robinson C. (2014). Children, Their Voices and Their Experiences of School: What Does the Evidence Tell Us?.

[20] Rettew D.C., Pawlowski S. (2016). Bullying. Child Adolesc. Psychiatr. Clin. N. Am..

[21] National Center for Educational Statistics (2019). Student Reports of Bullying: Results from the 2017 School Crime Supplement to the National Victimization Survey. US Department of Education. http://nces.ed.gov/pubsearch/pubsinfo.asp?pubid=2015056.

[22] Perren S., Ettekal I., Ladd G. (2013). The impact of peer victimization on later maladjustment: Mediating and moderating effects of hostile and self-blaming attributions. Child Psychol. Psychiatry.

[23] Brunstein Klomek A., Sourander A., Gould M. (2010). The association of suicide and bullying in childhood to young adulthood: A review of cross-sectional and longitudinal research findings. Canadian journal of psychiatry. Rev. Can. Psychiatr..

[24] Heydenberk R.A., Heydenberk W.R. (2017). Bullying reduction and subjective wellbeing: The benefits of reduced bullying reach far beyond the victim. Int. J. Wellbeing.

[25] Navarro R., Ruiz-Oliva R., Larrañaga E., Yubero S. (2015). The impact of cyberbullying and social bullying on optimism, global and school-related happiness and life satisfaction among 10-12-year-old schoolchildren. Appl. Res. Qual. Life.

[26] McCallion G., Feder J. (2013). Student Bullying: Overview of Research, Federal Initiatives, and Legal Issues.

[27] Davis S., Nixon C. (2010). The Youth Voice Research Project: Victimization and Strategies. http://njbullying.org/documents/YVPMarch2010.pdf.

[28] Trotman D., Tucker S., Martyn M. (2015). Understanding problematic pupil behaviour: Perceptions of pupils and behaviour coordinators on secondary school exclusion in an English city. Educ. Res..

[29] Ryan R., Deci E.L. (2001). On Happiness and Human Potentials: A Review of Research on Hedonic and Eudaimonic Well-Being. Annu. Rev. Psychol..

[30] Lau A.L.D., Cummins R.A., McPherson W. (2005). An Investigation into the Cross-Cultural Equivalence of the Personal Wellbeing Index. Soc. Indic. Res..

[31] Blanco A., Valera S. (2007). Los fundamentos de la intervención psicosocial. Interv. Psicosoc..

[32] López Hernáez L., Ovejero Bruna M. (2018). Percepción de las consecuencias del bullying más allá de las aulas: Una aproximación cuasi-cuantitativa. Pensam. Educ. Rev. De Investig. Latinoam. (PEL).

[33] Adegboyega L.O., Okesina F.A., Jacob O.A. (2017). Family Relationship and Bullying Behaviour among Students with Disabilities in Ogbomoso, Nigeria. Int. J. Instr..

[34] Miranda R., Oyanedel J., Torres J. (2018). Efectos del apoyo familiar, amigos y de escuela sobre el bullying y bienestar subjetivo en estudiantes de nivel secundario de Chile y Brasil. Apunt. Cienc. Soc..

[35] Konu A.I., Lintonen T.P., Autio V.J. (2002). Evaluation of well-being in schools–a multilevel analysis of general subjective well-being. Sch. Eff. Sch. Improv..

[36] Rigby K.E.N. (2000). Effects of peer victimization in schools and perceived social support on adolescent well-being. J. Adolesc..

[37] Nansel T.R., Overpeck M., Pilla R.S., Ruan W.J., Simons-Morton B., Scheidt P. (2001). Bullying behaviors among US youth: Prevalence and association with psychosocial adjustment. JAMA.

[38] Grills A.E., Ollendick T.H. (2002). Peer victimization, global self-worth, and anxiety in middle school children. J. Clin. Child Adolesc. Psychol..

[39] Nansel T.R., Craig W., Overpeck M.D., Saluja G., Ruan W.J. (2004). Cross-national consistency in the relationship between bullying behaviors and psychosocial adjustment. Arch. Pediatrics Adolesc. Med..

[40] Sánchez G., Blanco J.L. (2017). El «Buen trato», programa de prevención del acoso escolar, otros tipos de violencia y dificultades de relación: Una experiencia de éxito con alumnos, profesores y familia. Rev. De Estud. De Juv..

[41] Mäkelä T., López-Catalán B. (2018). Programa de convivencia y anti-acoso escolar finlandés KiVa. Impacto y reflexión. An. De La Fund. Canis Majoris.

[42] Seligman M. (2015). Evidence-Based Approaches in Positive Education: Implementing a Strategic Framework for Well-Being in Schools.

[43] Palomera R., Fernández-Abascal E.G. (2008). Educando para la felicidad. Emociones Positivas.

[44] Martín R.P. (2017). Psicología positiva en la escuela: Un cambio con raíces profundas. Pap. Del Psicólogo.

[45] Norrish J.M., Williams P., O’Connor M., Robinson J. (2013). An applied framework for positive education. Int. J. Wellbeing.

[46] Vives-Cases C., Davo-Blanes M.C., Ferrer-Cascales R., Sanz-Barbero B., Albaladejo-Blázquez N., Sánchez-San Segundo M., Lillo-Crespo M., Bowes N., Neves S., Mocanu V. (2019). Lights4Violence: A quasi-experimental educational intervention in six European countries to promote positive relationships among adolescents. BMC Public Health.

[47] Helliwell J., Layard R., Sachs J. (2015). World Happiness Report. https://s3.amazonaws.com/happiness-report/2015/WHR-2015-summary_final-ES.pdf.

